# miR-101 is down-regulated in glioblastoma resulting in EZH2-induced proliferation, migration, and angiogenesis

**DOI:** 10.18632/oncotarget.205

**Published:** 2011-01-03

**Authors:** Michiel Smits, Jonas Nilsson, Shahryar E. Mir, Petra M. van der Stoop, Esther Hulleman, Johanna M. Niers, Phillip C. de Witt Hamer, Victor E. Marquez, Jacqueline Cloos, Anna M. Krichevsky, David P. Noske, Bakhos A. Tannous, Thomas Würdinger

**Affiliations:** ^1^Neuro-oncology Research Group, Departments of Neurosurgery and Pediatric Oncology/Hematology, Cancer Center Amsterdam, VU University Medical Center, Amsterdam, the Netherlands; ^2^Molecular Neurogenetics Unit, Departments of Neurology and Radiology, Massachusetts General Hospital and Neuroscience Program, Harvard Medical School, Boston, MA, USA; ^3^Laboratory of Medicinal Chemistry, Center for Cancer Research, NCI-Frederick, MD, USA; ^4^Department of Neurology, Brigham and Women's Hospital, Harvard Medical School, Boston, MA, USA

**Keywords:** cancer, microRNA, Policomb group, glioblastoma, angiogenesis

## Abstract

**Background:**

Glioblastoma (GBM) is a malignant brain tumor with dismal prognosis. GBM patients have a median survival of less than 2 years. GBM is characterized by fast cell proliferation, infiltrative migration, and by the induction of angiogenesis. MicroRNAs and polycomb group (PcG) proteins have emerged as important regulators of gene expression.

**Methods:**

Here we determined that miR-101 is down-regulated in GBM, resulting in overexpression of the miR-101 target PcG protein EZH2, a histone methyltransferase affecting gene expression profiles in an epigenetic manner. Results: Inhibition of EZH2 *in vitro* by pre-miR-101, EZH2 siRNA, or small molecule DZNep, attenuated GBM cell growth, migration/invasion, and GBM-induced endothelial tubule formation. In addition, for each biological process we identified ontology-associated transcripts that significantly correlate with EZH2 expression. Inhibition of EZH2 *in vivo* by systemic DZNep administration in a U87-Fluc-mCherry GBM xenograft mouse imaging model resulted in reduced tumor growth.

**Conclusion:**

Our results indicate that EZH2 has a versatile function in GBM progression and that its overexpression is at least partly due to decreased miR-101 expression. Inhibition of EZH2 may be a potential therapeutic strategy to target GBM proliferation, migration, and angiogenesis.

## INTRODUCTION

GBM remains among the most devastating cancers with a median survival of less than 15 months and virtually no survival beyond five years [1]. GBM is the grade IV glioma and can arise *de novo* or through progression of lower grade gliomas. Evidence supporting the critical role of proliferation, migration and angiogenesis in the biological behavior of these tumors has led to a variety of studies on the basic mechanisms involved. GBM cells are highly proliferative but are also notorious because of their capacity to migrate through the brain parenchyma and their ability to induce angiogenic blood vessel sprouting. Several factors are involved in the angiogenesis process, which results in recruitment, proliferation and alignment of endothelial blood vessel cells through a complex interaction between endothelial cells and tumor cells [2].

miRNAs comprise a large group of endogenous non-coding RNAs that can block mRNA translation or negatively regulate mRNA stability and thereby play a central role in the regulation of gene expression [3]. It is also becoming clear that deregulated miRNA expression is a common feature of human diseases, especially in specific forms of cancer [4,5]. Recent studies have identified several miRNAs that are altered in GBM tumor cells themselves [6,7] as well as in GBM-associated endothelial cells [8].

PcG proteins are important epigenetic regulators which can function as transcriptional repressors that silence specific sets of genes through chromatin modification [9]. PcG proteins are grouped in polycomb repressive complexes (PRC). PRC2 includes enhancer of zeste 2 (EZH2), suppressor of zeste 12 (SUZ12), and embryonic ectoderm development (EED). EZH2 is the catalytically active component of PRC2 and is capable of trimethylating lysine 27 of histone H3 (H3K27) when in complex with SUZ12 and EED [10-15]. Recently, an increasing number of studies linked various oncogenic properties to EZH2, including impaired cellular differentiation and enhanced proliferation and *in vivo* tumor growth [16-22]. EZH2 is overexpressed in various cancers, which correlates to decreased patient survival [16,18,19,23-25]. Although EZH2 knock down was shown to be embryonic lethal in mice [26], knock down of EZH2 in cancer cells resulted in growth arrest, as well as in diminished tumor growth and reduced metastasis *in vivo* [16,20,22]. The role of PcG proteins in GBM is not well understood, but has been described to involve bone morphogenetic protein signaling, controlling the differentiation capacity of GBM cells [27].

Here we report that EZH2 expression in GBM is regulated by miR-101. We show that miR-101 is down-regulated in GBM cells, resulting in increased EZH2 expression and enhanced GBM cell proliferation, migration, and angiogenesis.

## RESULTS

**Figure 1:**
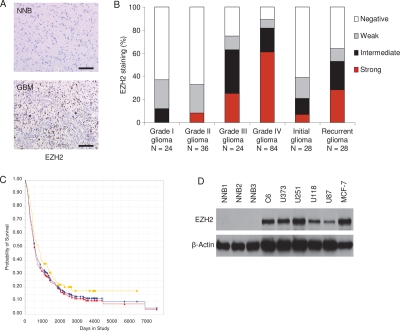
EZH2 expression is associated with high grade glioblastoma (A) Representative tissue sections stained with an antibody directed against EZH2. Immunohistochemical staining shows absent nuclear staining of non-neoplastic brain (NNB), and strong nuclear staining in glioblastoma (GBM). Scale bar = 100 µm. (B) Quantification of EZH2 protein expression in glioma tissue microarrays. Negative = 0%; Weak = <5%; Intermediate 5-25%; Strong = >25% positive EZH2 staining. EZH2 expression correlates to glioma grade (left) and to glioma recurrence (right). (C) Correlation between GBM patient survival and EZH2 mRNA expression, red indicates high EZH2 expression, yellow indicates low EZH2 expression and blue indicates all patients (http://caintegrator-info.nci.nih.gov/rembrandt). (D) EZH2 protein analysis by Western blot on various cell lines and non-neoplastic brain tissue (NNB).

To evaluate the expression levels of EZH2 in GBM cells and non-neoplastic brain (NNB) we performed immunohistochemistry for EZH2 protein expression on tissue microarrays containing GBM and NNB samples. Most of the GBM samples showed fields of strong nuclear staining for EZH2 while none of the NNB samples did (Fig. 1A). Increased EZH2 expression correlated with glioma grade and glioma recurrence (Fig. 1B), suggesting that EZH2 could be a marker for glioma aggressiveness. In addition, the Rembrandt database was used to show that EZH2 expression correlated with decreased GBM patient survival (Fig. 1C). EZH2 protein was strongly expressed in human GBM cell lines, including U251 and U87, but not in NNB (Fig. 1D).

In order to determine whether potential GBM-expressed miRNAs could affect EZH2 expression we first determined which miRNAs expressed in NNB are differentially expressed in GBM (Supplemental Table S1A). Next, we used miRbase [28] to identify 63 miRNAs predicted to target EZH2. Upon integration of the list of miRNAs predicted to target EZH2 and the differential GBM/NNB miRNA expression ratios, we found that miR-101, miR-98, miR-137, and miR-139 were down-regulated in GBM tissue as compared to NNB and have the potential to regulate EZH2 (Supplemental Table S1B). Another miRNA which was previously found to target EZH2, miR-26a [29], was not included in our subset of miRNAs expressed in the brain, and therefore not part of our study.

**Figure 2:**
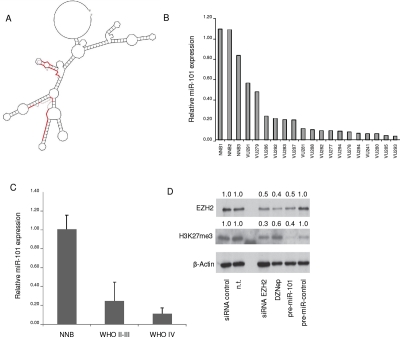
miR-101 is down-regulated in GBM and targets EZH2 (A) Predicted RNA structure of the 3'UTR of EZH2 by RNAfold software, in red the 2 miR-101 biniding sites are indicated. (B and C) Down-regulation of miR-101 was confirmed by qRT-PCR analysis. RNA extracted from surgically removed gliomas from patients was analyzed by qRT-PCR. The data were normalized to the level of GAPDH mRNA in each sample. NB = Non-neoplastic brain; II, III, IV indicate WHO glioma grades. (D) EZH2 and H3K27me3 expression analysis by Western blot in U87 GBM cells following transfection with pre-miR-101, pre-miR-control, EZH2 siRNA or control, or treatment with DZNep. Data presented as relative EZH2 expression compared to non-transfected (n.t.) cells. Error bars indicate s.d. *p<0.05, ***p<0.001, t test.

We were particularly interested in miR-101 since it was confirmed to bind the EZH2 3’-UTR at two sites (Fig. 2A), and was recently shown to interact with EZH2 in other types of cancer [30,31]. Previous analysis showed that genomic loss of miR-101-1 and miR-101-2 alleles was observed in 18.7% of GBM cases [30,32]. Based on these findings, we decided to further analyze miR-101/EZH2 functionality in GBM. First, down-regulation of miR-101 was confirmed in primary glioma samples of different WHO grades by quantitive PCR (qRT-PCR) analysis (Fig. 2B and 2C). To establish that miR-101 affects EZH2 protein expression and histone methyltransferase activity in GBM, we transfected human U87 GBM cells with pre-miR-101 molecules and determined the levels of EZH2 protein and H3K27me3. In addition to pre-miR-101, we included EZH2 siRNA, non-related control oligonucleotides, and the S-adenosylhomocysteine hydrolase inhibitor DZNep, a potent EZH2 inhibitor [33,34]. DZNep, EZH2 siRNA, and pre-miR-101 all notably repressed EZH2 protein expression and reduced the levels of trimethylation of H3K27 (Fig. 2D), indicating inhibition of EZH2 function.

**Figure 3:**
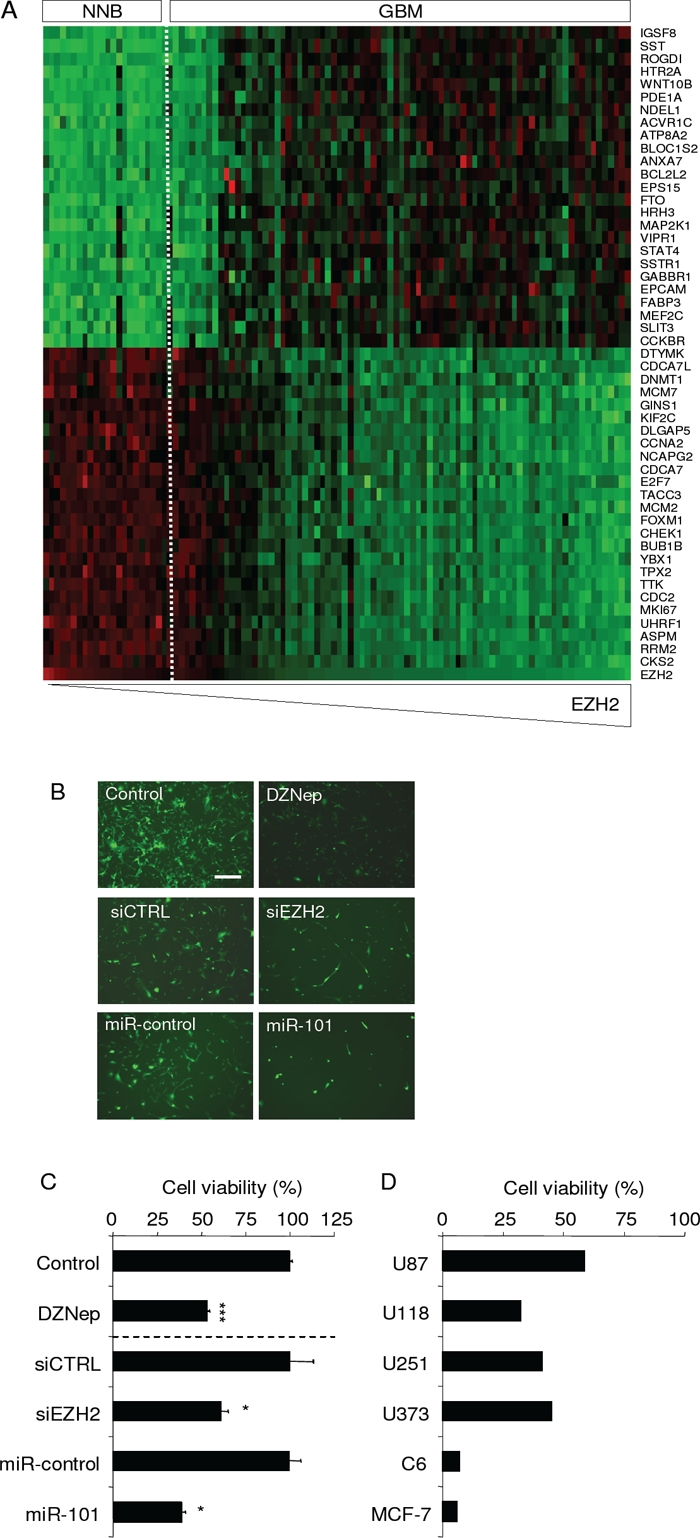
miR-101-regulated EZH2 affects GBM proliferation *in vitro* (A) *In silico* analysis of EZH2 mRNA expression and the correlation to proliferation-related mRNAs. Heatmap of percentile fold change of gene expression of proliferation-related genes sorted by correlation with EZH2 expression (rows) in patients sorted by level of EZH2 expression (columns). (B) Fluorescence microscopical images of U87-GFP cells 96 h after inhibition of EZH2 by transfection with miR-101 precursor, EZH2 siRNA or treatment with DZNep. Scale bar = 450 µm. (C) U87-GFP cells were treated as in (B) and cell proliferation was measured by WST-1 proliferation assay. (D) A panel of human GBM cell lines, (U87, U118, U251 and U373) and a rat GBM cell line (C6) were treated with 5 µM DZNep. Proliferation compared to untreated control was measured by cell counts in a casy count apparatus. Human breast cancer cell line MCF-7 was included as a positive control. Error bars indicate s.d. *p<0.05, ***p<0.001, t test.

To determine the effects of EZH2 on GBM cell proliferation we first analyzed which genes associated with cell proliferation correlated with EZH2 expression in GBM and NNB [35]. First, EZH2 was found overexpressed in most GBM samples as compared to NNB. However, in few samples the EZH2 mRNA expression was found to be in the same range as in the NNB (Fig. 3A). Out of the 1419 genes that were linked to the proliferation gene ontology as determined by AmiGO [36], 214 genes showed a clear correlation (>67%) with EZH2 expression in GBM (Fig. 3A). Interestingly, the GBM samples with normal EZH2 expression levels also showed less expression of the genes associated with cell proliferation. Next, cellular proliferation was studied in GBM cell cultures to determine if EZH2 influences the proliferation of GBM cells. miR-101 induction, EZH2 knock down by siRNA and treatment with DZNep significantly reduced cellular proliferation in U87-GFP GBM cells (Fig. 3B and 3C). The effect of DZNep treatment on proliferation inhibition was confirmed in other GBM cell lines *in vitro* (Fig. 3D).

**Figure 4:**
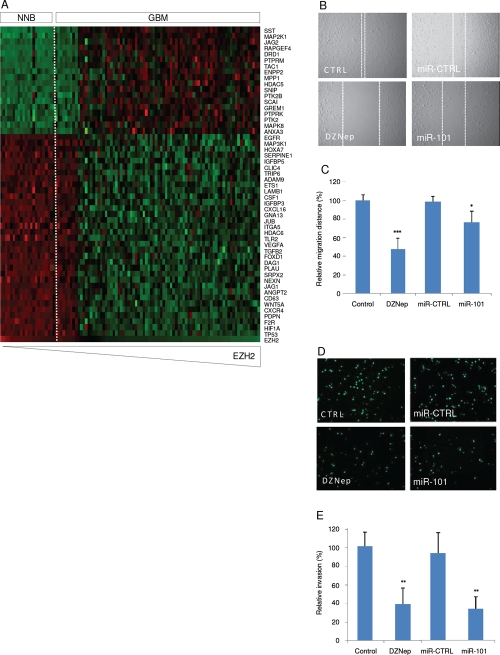
*In silico* analysis of EZH2 mRNA expression and the correlation to migration-related mRNAs Heatmap of percentile fold change of gene expression of migration-related genes sorted by correlation with EZH2 expression (rows) in patients sorted by level of EZH2 expression (columns). Color coding is similar to Fig 3A. (B) U87 monolayer cultures were scratched. Images were acquired directly after scratching (t = 0) and 24 h later (t = 24). The migration front is indicated by the dashed lines. Scale bar = 450 µm. (C) Quantitation of cell migration into the scratch using ImageJ software. (E and F) U87 cells were transfected with pre-miR-101, EZH2 siRNA, non-related control molecules, or treated with DZNep, and analyzed for invasion capability. EZH2 inhibition decreased invasion as shown by Hoechst staining. Error bars indicate s.d. *p<0.05, ***p<0.001, t test. Scale bar = 225 µm.

To determine the effects of EZH2 on GBM cell migration we analyzed which genes belonging to the migration gene ontology correlated with EZH2 expression in GBM and NNB. A significant correlation between the expression of 28 out of 279 genes associated with cell migration and EZH2 expression was observed (Fig. 4A). In order to determine whether miR-101 up-regulation or EZH2 inhibition also affected GBM cell migration, scratch assays were performed. Up-regulation of miR-101 by pre-miR-101 resulted in a significant decrease in U87 migration. The EZH2 inhibitors DZNep and EZH2 siRNA showed a similar decrease in migration (Fig. 4B and 4C). To further evaluate the effects of miR-101/EZH2 modulation on *in vitro* migration and invasion, a Boyden chamber assay was used. U87 cells that were transfected with pre-miR-101 showed a significant decrease in ability to invade, as visualized by Hoechst staining (Fig. 4D and quantitated in 4E). Again, similar results were observed after treatment with the EZH2 inhibitor DZNep or EZH2 knock down by siRNA (Fig. 4D and 4E).

**Figure 5:**
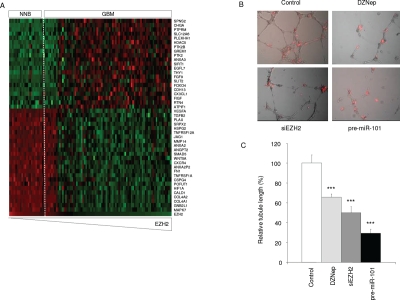
miR-101/EZH2 affects GBM angiogenesis *in vitro* (A) In silico analysis of EZH2 mRNA expression and the correlation to angiogenesis-related mRNAs. Heatmap of percentile fold change of gene expression of angiogenesis-related genes sorted by correlation with EZH2 expression (rows) in patients sorted by level of EZH2 expression (columns). Color coding is similar to Fig 3A. (B) U87-Fluc-mCherry cells were treated and co-cultured with HBMVECs on Matrigel coated plates. Tubule formation was assessed 96 h after transfection as tubule length and branching. Scale bar = 450 µm. (C) Quantitation of tubule length and branching in (B) using ImageJ software. Error bars indicate s.d. *p<0.05, ***p<0.001, t test.

To determine the effects of EZH2 on GBM-induced angiogenesis we also analyzed which genes belonging to the angiogenesis gene ontology correlated with EZH2 expression in GBM. Again, a significant correlation between the expression of 33 out of 308 genes associated with angiogenesis and EZH2 expression was observed (Fig. 5A). Next, HBMVECs were cultured in EBM, EGM, or EBM supplemented with U87 human GBM cells expressing GFP (U87-GFP), all on a Matrigel substratum to promote tubule network formation. Tubules were visualized by a combination of light and fluorescence microscopy. After pre-treatment of the GBM cells with DZNep, or transfection with pre-miR-101, EZH2 siRNA, or non-related oligonucleotides of similar chemistry, and subsequent co-culturing with HBMVECs on Matrigel, we analyzed tubule length and tubule branching. Up-regulation of miR-101 in U87-GFP cells resulted in a substantial decrease in total tubule length and tubule branching (Fig. 5B and 5C). In addition to pre-miR-101, treatment of the U87-GFP cells with EZH2 siRNA and DZNep also inhibited U87-induced tubule network formation (Fig. 5B and 5C).

**Figure 6:**
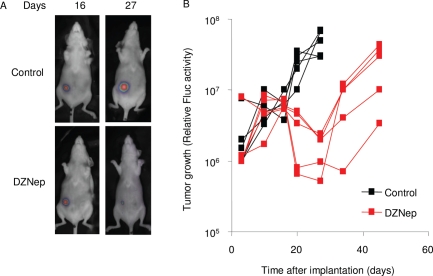
Inhibition of EZH2 affects GBM development *in vivo* (A) 1 × 10^6^ U87-Fluc-mCherry cells were implanted s.c. in nude mice. Tumor growth was monitored by *in vivo* Fluc bioluminescence imaging. After implantation, one set of mice was injected i.v. with DZNep and another set with PBS, at day 3, 5 and 7, followed by weekly injection. (B) Quantitation of Fluc.

Finally, to study the effects of modulation of EZH2 on GBM growth *in vivo*, we implanted U87 human GBM cells stably expressing Fluc and the fluorescent protein mCherry (U87-Fluc-mCherry) into the flanks of nude mice. Tumor growth was monitored over time by intravenous injection of the Fluc substrate D-luciferin and *in vivo* bioluminescence imaging using a CCD camera. After tumor cell implantation, we injected one set of mice (n = 5) intravenously with the EZH2 inhibitor DZNep (0.07 mg/kg) and a parallel control set (n = 5) with PBS only, at day 3, 5, and 7, followed by weekly injection. CCD camera imaging of Fluc bioluminescence activity in the tumor allowed us to monitor tumor growth over time. The tumor volume of the mice treated with PBS increased logarithmically over time, while the tumor volume in the mice treated with DZNep showed reduced growth (Fig. 6A and 6B).

## DISCUSSION

Here we show that miR-101 is down-regulated in glioma in a grade dependent manner. The impaired translational repression of EZH2 by miR-101 causes EZH2 overexpression in GBM, which correlates with patient survival. EZH2 is a methyltransferase that affects the expression of many genes. Based on *in silico* expression analysis and EZH2 expression correlation we found that EZH2 overexpression induces glioma proliferation, migration/invasion, and angiogenesis, processes driving glioma progression.

miR-101/EZH2 was found to be deregulated in several other types of cancer, including prostate cancer [30], bladder transitional cell carcinoma [31], gastric cancer [37], and described to strongly correlate with migration, invasion, and metastasis [30,37]. Here we demonstrate a role for EZH2 overexpression in GBM, which could be inhibited by miR-101, siEZH2, or small molecule inhibitor DZNep. Besides induction of migration and invasion, we identified a role for EZH2 in cellular proliferation and the induction of angiogenesis, indicating a versatile pro-tumoral function for EZH2 in GBM. Inhibition of EZH2 by DZNep resulted in reduced GBM growth *in vitro* and in a limited experiment *in vivo*. Altogether, these results indicate that EZH2 may be a useful drug target for the treatment of GBM.

miRNAs are know to affect cellular processes such as angiogenesis [8,38,39]. Here we show a role for miR-101 in GBM-induced angiogenesis. Unpublished data indicate that miR-101 also regulates EZH2 in endothelial cells. We provide evidence that miR-101 down-regulation regulates angiogenesis by induction of EZH2 and a pro-angiogenic mRNA profile. However, the exact mechanisms of EZH2 function in endothelial and GBM cells remains to be investigated. Besides miR-101, we also found the predicted EZH2 targeting miRNAs miR-98, miR-137, and miR-139 to be down-regulated in GBM cells as compared to NNB tissue. It remains to be investigated whether these miRNAs truly repress EZH2, and whether other previously identified miR-101 target genes are also repressed by miR-101 in GBM cells, these may include Cox-2, Mcl-1 and Fos [37], MAGI-2 [40], DNA-PKcs and ATM [41], COX-2 [42]. The complex interaction of reduced miR-101-mediated translational repression and increased EZH2-mediated transcriptional repression at least seem to cause pro-tumoral switches in the GBM transcriptome profile. Interestingly, our results also identified that a subset of GBMs express normal levels of EZH2 mRNA. We found that the genes associated with proliferation, migration, and angiogenesis were also expressed in the normal range, following the EZH2 expression levels. Further research in the nature and behavior of this subset of EZH2-low GBMs and its correlation to miR-101 expression is warranted.

In conclusion, our results indicate that EZH2 has a versatile pro-tumoral function in GBM and that its overexpression is at least partly due to decreased miR-101 expression. Inhibition of EZH2 may be a potential therapeutic strategy to target GBM proliferation, migration, and angiogenesis.

## MATERIALS AND METHODS

### Cells

Human brain microvascular endothelial cells (HBMVECs; Cell Systems ACBRI-376) were cultured in EGM medium (Lonza). C6, 293T, MCF-7, U118, U251, U373, and U87 cells (U-87 MG; ATCC) were cultured in DMEM (Lonza) containing 10% FBS and antibiotics. U87-Fluc-mCherry cells were produced by stably transducing U87 cells with CMV-controlled expression cassette using a lentiviral vector [43].

### Quantitive RT-PCR

Quantitive RT-PCR (qRT-PCR) analysis was used to determine the relative expression levels of miR-101, miR-186, EZH2, and GAPDH mRNA. Total RNA was isolated using the miRVANA miRNA isolation kit. Equal amounts of RNA were converted into cDNA using miR-101, miR-186, EZH2, and GAPDH RT primers (Applied Biosystems and Qiagen, according to the manufacturer's protocol). Subsequently, quantitive PCR was performed using primers and materials from Applied Biosystems. All experiments were performed using biological triplicates and experimental duplicates. The data was normalized to miR-186 or GAPDH expression levels.

### Disclosed primers used were:

human EZH2 (forward)/ (reverse)

5’-CCTGAAGTATGTCGGCATCGAAAGAG-3’

5’-TGCAAAAATTCACTGGTACAAAACACT-3’

human GAPDH (forward)/ (reverse)

5’-GTCGGAGTCAACGGATT-3’

5’-AAGCTTCCCGTTCTCAG-3’

### 2.3 Western blots and immunohistochemistry

EZH2 protein and H3K27me3 expression was detected by SDS-PAGE followed by Western blot analysis, using mouse anti-EZH2 monoclonal antibody (BD biosciences) and rabbit anti-H3K27me3 (Upstate Biotechnology). Mouse anti-Actin (Millipore) was used as a loading control. Protein levels were detected using ECL detection solution (GE healthcare) and visualized on X-ray film (GE healthcare). Paraffin sections of human GBM tissue and NNB were incubated with monoclonal mouse anti-EZH2 (BD biosciences) antibodies. Positive reactions were visualised using a secondary antibody (DAKO EnvisionHRP) and 3,3-diaminobenzidine or liquid red chromogen.

### miRNA modulation

50 nM of pre-miR-101 (Ambion) or pre-miR-control (Ambion) oligonucleotides were transfected into U87 human GBM cells using Lipofectamin2000 (Invitrogen), according the manufacturer's protocol. For the inhibition of EZH2 50 nM of EZH2 siRNA (Qiagen) was transfected into U87 cells. siRNA-AF or control oligonucleotides (Qiagen) were used as controls. After 5 h of transfection, the transfection medium was replaced by DMEM until further analysis.

### In silico analysis

A RNA microarray dataset containing 81 GBM and 23 NNB samples as published by [35] was used to obtain mRNA expression estimates. To quantify the differential level of expression for each glioblastoma sample, the significance analysis of microarrays algorithm was performed using the samr package (version 1.24 by B. Narasimhan and R. Tibshirani) in R, A Language and Environment for Statistical Computing (release 2.4.1; Vienna, Austria; http://www.R-project.org) based on randomization. Unpaired two class comparison for cancer versus normal tissue samples was performed, unless paired samples were involved, in which case a paired analysis was performed. The selection of the delta parameter was based on a median false discovery rate less than 0.05. The default number of 100 permutations was used. This resulted in fold change values for all 19769 genes on the Affymetrix microarray platform. Using the AmiGO tool [36] of the Gene Ontology project [44] lists of transcripts associated with the biological processes proliferation (GO:0008283; 9/20/10), migration (GO:0030334; 9/20/10) and angiogenesis (GO:0001525; 9/20/10) were obtained. Lists of unique transcripts were prepared by removing duplicate entries. Subsequently the microarray dataset was queried for the genes in each of these ontologies. Samples were sorted on EZH2 expression level for the NNB and GBM samples separately and the average expression levels scaled on a gene by gene basis for genes significantly correlating with EZH2 expression (absolute value Pearson correlation > 0.667) were plotted as a heatmap using the gplots package in R.

### In vitro angiogenesis assay

HBMVECs were cultured on Matrigel (BD biosciences) in EBM (Lonza) in the presence or absence of U87-Fluc-mCherry cells, or EGM (Lonza). The experiments were performed in triplicate, repeated twice and judged in a double blind fashion by at least two observers. At least 3 pictures were taken randomly of each culture well using a digital camera system (Leica). Total tubule length and number of branches were analyzed using the software program ImageJ.

### In vitro migration assay

GBM cells were grown to confluence in 24-wells plates and were transfected with 50 nM of oligonucleotides or treated with DZNep (5 µM). At 72 h after transfection an artificial wound was created using a pipette tip after which the cells were further incubated. To analyse cell migration into the scratched area, pictures were taken at 0 and 24 h using a digital camera system coupled to a microscope. ImageJ was used to determine the migration distance (in μm) as the reduction of the width of the open area.

### In vitro invasion assay

GBM invasion function was analyzed using a Boyden chamber assay. A 24-well Transwell system (Corning) was used, with each well containing a permeable transwell insert containing a 6.5 mm polycarbonate membrane with 8 μm pores. The inserts were coated with 4x diluted basement membrane extract (Trevigen) and incubated overnight in 5% CO_2_ at 37°C. GBM cells were transfected with 50 nM of oligonucleotides for 24 h or treated with DZNep (5 µM for 24 h), starved for 24 h and harvested in serum free medium. Per insert 25,000 GBM cells were subsequently placed on the membrane. The inserts were immersed in a 24-well plate that was filled with EGM growth medium culture media. After incubation for 24 h, the membrane was washed briefly with PBS. The upper side of the membrane was then wiped gently with a cotton ball. The membrane was then fixed in 4% formaldehyde and stained with Hoechst. The magnitude of GBM cell migration was evaluated by counting the migrated cells in 3 random high-power (5x) microscope fields.

### Tumor model and bioluminescence imaging

All animal studies were approved by the Massachusetts General Hospital Review Board. Nude mice were anesthetised with i.p. injection of xylazine (5 mg/kg) and ketamine (100 mg/kg). 50 µl containing 1 × 10^6^ U87-Fluc-mCherry cells were pre-mixed with an equal volume of matrigel (BD biosciences) and implanted in the flanks of nude mice. DZNep was administered intravenously to tumor-bearing mice at a dose of 0.07 mg/kg diluted in 100 µl PBS at day 3, 5 and 7 after tumor implantation, followed by weekly injection. Mice were anesthetized as above and Fluc imaging was performed 10 min after intravenous injection of 150 µl beetle D-luciferin (4 mg/kg body weight) (Xenogen), and recording photon counts over 5 min using a cooled CCD camera with no illumination. Dim polychromatic illumination was used to take a light image of the animal. Visualization was performed using CMIR-Image, a program developed by the Center for Molecular Imaging Research using image display and analysis suite developed in IDL (Research Systems Inc., Boulder, CO). An intensity contour procedure to identify bioluminescence signals with intensities significantly greater than the background was used to define regions of interest. The mean, standard deviation and sum of photon counts in these regions were calculated as a measurement of Fluc activity.

### Statistics

Difference in biological properties between parental and EZH2 downregulated cells was analysed using Student's t-test. P-values <0.05 were considered statistically significant.

## SUPPLEMENTAL TABLES

Supplemental Table S1a

Supplemental Table S1b
