# Supplementary material for: miR-101 is down-regulated in glioblastoma resulting in EZH2-induced proliferation, migration, and angiogenesis
Source: Oncotarget. 2011 Jan 3;1(8):710–20. doi: 10.18632/oncotarget.205 (PMC3124376; doi:10.18632/oncotarget.205)
Supplement: Supplemental Table S1a [file oncotarget-01-710-s001.pdf]

Supplemental Table S1a. Table of deregulated miRNAs in GBM that potentially regulate EZH2. A selection of miRNAs expressed in the brain was analyzed for its differential expression in non-neoplastic brain (NNB) and glioblastoma (GBM) tissues, using qRT-PCR (Applied Biosystems, according the manufacturer's protocol) [45]. miRNAs that were found to be differentially expressed in GBM (GBM/NNB ratio <0.65 or >1.70) and are predicted to target the EZH2 3'-UTR are indicated.

miRNA	Fold change (GBM/NNB)	
miR-21	4.997136	Up
miR-16	0.667656	
miR-15b	1.998466	Up
miR-15a	1.507183	
miR-92	2.505202	Up
miR-19a	1.474438	
miR-19b	0.587649	Down
miR-17-5p	3.680952	Up
miR-128a	0.655279	
miR-7	0.071265	Down
miR-1	0.256097	Down
miR-29a	0.5813	Down
miR-29b	0.523982	Down
miR-29c	1.165094	
miR-134	0.286395	Down
miR-30a-5p	0.287311	Down
miR-22	0.243365	Down
miR-96	1.631808	
miR-182*	1.807229	Up
miR-183	37.37386	Up
miR-219	0.016776	Down
miR-139	0.00975	Down
miR-330	0.02896	Down
miR-124a	0.02607	Down

miRNA	Fold change (GBM/NNB)	
miR-338	0.193976	Down
miR485-5p	0.030904	Down
miR-181a	0.008061	Down
miR-20	1.451454	
miR-98	0.527855	Down
miR-200a	0.226573	Down
miR-324-3p	0.0587	Down
miR-378	0.246587	Down
miR-103	0.018079	Down
miR-467	1.03631	
miR-521	1	
miR-126*	0.350301	Down
miR-155	1.153513	
miR-137	0.00616	Down
miR-30d	0.064749	Down
miR-135a	0.051153	Down
miR-101	0.601473	Down
