# Supplementary material for: miR-101 is down-regulated in glioblastoma resulting in EZH2-induced proliferation, migration, and angiogenesis
Source: Oncotarget. 2011 Jan 3;1(8):710–20. doi: 10.18632/oncotarget.205 (PMC3124376; doi:10.18632/oncotarget.205)
Supplement: Supplemental Table S1b [file oncotarget-01-710-s002.pdf]

Supplemental Table S1b. The miRNA alignments in the table are based on miRNAs predicted to regulate the 3'UTR of EZH2 using miRBase (http://microrna.sanger.ac.uk/targets/v5/). The 3'UTR miRNA binding sites of EZH2 are presented and extracted from the Ensemble database (ENST00000320356). Of the miRNAs deregulated in GBM, only miR-101, miR-98, miR-137 and miR-139 were predicted to target the 3'-UTR of EZH2. miR-101 showed the highest binding predictions and was predicted to bind the EZH2 3'-UTR twice.

<table>
<tr><th>miRNA ID</th><th>Score</th><th>Start</th><th>End</th><th>Alignment miRNA
3'UTR</th><th>Species</th><th>No. hits within 3'-UTR</th><th>Confirmed deregulated in GBM</th></tr>
<tr><td>hsa-miR-101</td><td>18,8608</td><td>101</td><td>121</td><td>AGUCAAUAGUGUCAUGACAU
| | || || |||||||||
TGAATTTGCAAAGTACTGTA</td><td>7</td><td>2</td><td>Down</td></tr>
<tr><td>hsa-miR-127-5p</td><td>18,761</td><td>28</td><td>49</td><td>uagUCUCGGGAGACUCGAAGUC
   | |||: || ||||||||
gaaACAGCTGCCTTAGCTTCAG</td><td>1</td><td>1</td><td></td></tr>
<tr><td>hsa-miR-101</td><td>18,6322</td><td>45</td><td>66</td><td>AAGUCAAUAGUG-UCAUGACAU
||||| | | | ||||||||:
TTCAGGAACCTCGAGTACTGTG</td><td>8</td><td>2</td><td>Down</td></tr>
<tr><td>hsa-miR-23a*</td><td>18,5336</td><td>35</td><td>56</td><td>uuuaGGGUAGGGGUCCUUGGGG
    |::| |::||||||||:|
ctgcCTTAGCTTCAGGAACCTC</td><td>2</td><td>1</td><td></td></tr>
<tr><td>hsa-let-7f</td><td>18,4199</td><td>1</td><td>14</td><td>uugauauguUAGAUGAUGGAGu
         ||||:|||||||
--------cATCTGCTACCTCc</td><td>1</td><td>1</td><td></td></tr>
<tr><td>hsa-miR-765</td><td>18,2893</td><td>1</td><td>18</td><td>guaGUGGAAGGAAGAGGAGGu
   ||:|| |: |||||||
---CATCTGCTACCTCCTCCc</td><td>2</td><td>1</td><td></td></tr>
<tr><td>hsa-let-7a</td><td>18,0788</td><td>1</td><td>14</td><td>uugauauguUGGAUGAUGGAGu
         |:||:|||||||
--------cATCTGCTACCTC</td><td>1</td><td>1</td><td></td></tr>
<tr><td>hsa-let-7b</td><td>18,0788</td><td>1</td><td>14</td><td>uuggugugguUGGAUGAUGGAGu
         |:||:|||||||
--------cATCTGCTACCTCc</td><td>1</td><td>1</td><td></td></tr>
<tr><td>hsa-let-7c</td><td>18,0788</td><td>1</td><td>14</td><td>uugguauguUGGAUGAUGGAGu
         |:||:|||||||
--------cATCTGCTACCTCc</td><td>1</td><td>1</td><td></td></tr>
<tr><td>hsa-let-7d</td><td>18,0788</td><td>1</td><td>14</td><td>uugauacguUGGAUGAUGGAGa
         |:||:|||||||
--------cATCTGCTACCTCc</td><td>1</td><td>1</td><td></td></tr>
<tr><td>hsa-miR-217</td><td>17,8748</td><td>193</td><td>220</td><td>aGGUUAGUCAAGGAC-----UACGUCAU
 |||:| | ||::|| ||||||||
aCCAGTGAATTTTTGCAATAATGCAGTA</td><td>5</td><td>2</td><td></td></tr>
<tr><td>hsa-miR-139-5p</td><td>17,8514</td><td>47</td><td>68</td><td>GACCUCUGUGCACGUGACAUCU
| ||| | || |:|||||:|:
CAGGAACCTCGAGTACTGTGGG</td><td>7</td><td>1</td><td>Down</td></tr>
<tr><td>hsa-miR-19b-2*</td><td>17,8514</td><td>94</td><td>116</td><td>ACUUUACG-UUUGGACGUUUUGa
|||||| | :||::||||||::
TGAAATTCTGAATTTGCAAAGTa</td><td>1</td><td>1</td><td></td></tr>
<tr><td>hsa-miR-630</td><td>17,8514</td><td>231</td><td>253</td><td>uGGAAGGGAC-CAUGUCUUAUGA
 :| |::|| || ||||||||
tTCAACTTTGAATAAAGAATACT</td><td>6</td><td>1</td><td></td></tr>
<tr><td>hsa-miR-20a*</td><td>17,624</td><td>198</td><td>219</td><td>gaaauucacgagUAUUACGUCA
            ||||||||||
tgaattttgcaATAATGCAGT</td><td>3</td><td>1</td><td></td></tr>
<tr><td>hsa-let-7e</td><td>17,5103</td><td>1</td><td>14</td><td>uugauauguUGGAGGAUGGAGu
         |:|| |||||||
--------cATCTGCTACCTCc</td><td>1</td><td>1</td><td></td></tr>
<tr><td>hsa-let-7g</td><td>17,5103</td><td>1</td><td>14</td><td>uugacauguUUGAUGAUGGAGu
         | ||:|||||||
--------cATCTGCTACCTCc</td><td>1</td><td>2</td><td></td></tr>
<tr><td>hsa-let-7i</td><td>17,5103</td><td>1</td><td>14</td><td>uugucguguUUGAUGAUGGAGu
         | ||:|||||||
--------cATCTGCTACCTCc</td><td>1</td><td>1</td><td></td></tr>
<tr><td>hsa-miR-595</td><td>17,4891</td><td>211</td><td>231</td><td>ucUGUGUGGUGCCGUGUGAAG
  |::|| :|:||:|||:||:
taATGCAGTATGGTACATTTT</td><td>6</td><td>1</td><td></td></tr>
<tr><td>hsa-miR-26a</td><td>17,2829</td><td>236</td><td>257</td><td>ucggauaggacCUAAUGAACUU
           || ||||||||
ctttgaataaaGAATACTTGAA</td><td>7</td><td>1</td><td></td></tr>
</table>

<table>
<tr><td>hsa-miR-150*</td><td>17,1692</td><td>176</td><td>197</td><td>gacagggguCCGGACAUGGUC
          | ::||||||||
gctgcaaagtGTTTTGTACCAG</td><td>2</td><td>1</td><td></td></tr>
<tr><td>hsa-miR-98</td><td>17,1692</td><td>1</td><td>14</td><td>uuguuauguUGAAUGAUGGAGu
         |: |:|||||||
--------cATCTGCTACCTCc</td><td>1</td><td>1</td><td>Down</td></tr>
<tr><td>hsa-miR-944</td><td>17,0555</td><td>109</td><td>131</td><td>gaguaggcuACA-UGUUAUUAAA
         ||| | ||||||||
gcaaagtacTGTAAGAATAATTT</td><td>7</td><td>1</td><td></td></tr>
<tr><td>hsa-miR-26b</td><td>17,0319</td><td>237</td><td>257</td><td>uggauaggacUUAAUGAACUU
          :| ||||||||
tttgaataaaGAATACTTGAA</td><td>7</td><td>1</td><td></td></tr>
<tr><td>hsa-miR-539</td><td>16,9418</td><td>213</td><td>234</td><td>UGUGUGGUUCCUAUUAAAGAGg
|::|| :| || ||||:||
ATGCAGTATGGTACATTTTTCa</td><td>4</td><td>1</td><td></td></tr>
<tr><td>hsa-miR-646</td><td>16,8821</td><td>22</td><td>40</td><td>cGGAGUCUCCGUCGACGaa
 |||| || |||||||
tCCTCTGAAACAGCTGCct</td><td>3</td><td>1</td><td></td></tr>
<tr><td>hsa-miR-498</td><td>16,8566</td><td>76</td><td>98</td><td>CUUUUUGCGGGGGACCGAACUUU
|||||| | : |:||||||
GAAAAAGAACATGCAGTTTGAAA</td><td>5</td><td>1</td><td></td></tr>
<tr><td>hsa-miR-33a</td><td>16,8033</td><td>198</td><td>218</td><td>ACGUUACGUUGAUGUUACGUg
|| | | : :| |:||||||
TGAATTTTTGCAATAATGCAg</td><td>6</td><td>1</td><td></td></tr>
<tr><td>hsa-miR-330-3p</td><td>16,7435</td><td>168</td><td>192</td><td>AGAGACGUC--CGGCACACGAAACG
|||| ||| || |||:||||:
TCTCACCAGCTGCAAAGTGTTTTGT</td><td>4</td><td>1</td><td></td></tr>
<tr><td>hsa-miR-27a</td><td>16,6889</td><td>47</td><td>68</td><td>cgCCUUGAAUC-GGUGACACUU
  ||||| | | :||||||::
caGGAACCTCGAGTACTGTGGG</td><td>6</td><td>1</td><td></td></tr>
<tr><td>hsa-miR-138</td><td>16,6303</td><td>154</td><td>178</td><td>gccGGACUAA--GUGUUGUGGUCGA
   ::| ||| | : ||||||||
actTTTTATTGCCTTCTCACCAGCT</td><td>8</td><td>1</td><td></td></tr>
<tr><td>hsa-miR-99a*</td><td>16,6007</td><td>125</td><td>146</td><td>gucUGGGUAUCUUCGCUCGAAc
   |:::|||| | :|||:||
ataATTTATAGTAATGAGTTTa</td><td>2</td><td>1</td><td></td></tr>
<tr><td>hsa-miR-20a</td><td>16,5172</td><td>53</td><td>75</td><td>gauggaCGUGAUAUUCGUGAAAU
      |:|||:|::||| ||||
cctcgaGTACTGTGGGCAATTTA</td><td>5</td><td>1</td><td></td></tr>
<tr><td>hsa-miR-219-1-3p</td><td>16,487</td><td>137</td><td>158</td><td>gccCUGCAGGUCUGAGUUGAGA
   || |: | | ||||||:|
aatGAGTTTAAAAATCAACTTT</td><td>2</td><td>2</td><td></td></tr>
<tr><td>hsa-miR-23b*</td><td>16,487</td><td>37</td><td>56</td><td>uuuAGUCGUACGGUCCUUGGGu
   |:||| | ||||||||:
gccTTAGCTT--CAGGAACCTc</td><td>2</td><td>1</td><td></td></tr>
<tr><td>hsa-miR-106b</td><td>16,4603</td><td>55</td><td>75</td><td>uagaCGUGACAGUCGUGAAAU
    |:||||| :||| ||||
tcgaGTACTGTGGGCAATTTA</td><td>5</td><td>1</td><td></td></tr>
<tr><td>hsa-miR-128a</td><td>16,4603</td><td>47</td><td>67</td><td>uUUCUCUGGCCAAGUGACACU
 |:|| |: | :||||||:
cAGGAACCTCGAGTACTGTGG</td><td>6</td><td>1</td><td></td></tr>
<tr><td>hsa-miR-871</td><td>16,4041</td><td>85</td><td>107</td><td>GUACUGACCGUGAUUAGACUUAu
|||| :: | |||||||
CATGCAGTTTGAAATTCTGAATt</td><td>5</td><td>1</td><td></td></tr>
<tr><td>hsa-miR-382</td><td>16,3733</td><td>56</td><td>75</td><td>GCUUAGGUGGUGCUUGUUGAAg
|||:| ||:::|::|||:||
CGAGT--ACTGTGGGCAATTTa</td><td>1</td><td>2</td><td></td></tr>
<tr><td>hsa-miR-27b</td><td>16,346</td><td>47</td><td>68</td><td>cgUCUUGAAUC-GGUGACACUU
  :|||| | | :||||||::
caGGAACCTCGAGTACTGTGGG</td><td>6</td><td>1</td><td></td></tr>
<tr><td>hsa-miR-33b</td><td>16,3227</td><td>199</td><td>218</td><td>CGUUACGUUGUCGUUACGUg
| | | : :|| :||||||
GAATTTTTGCAATAATGCAg</td><td>6</td><td>1</td><td></td></tr>
<tr><td>hsa-miR-452</td><td>16,2596</td><td>16</td><td>36</td><td>agucaaAGGAGACGUUUGUCaa
      ||||||| ||||||
ccccccTCCTCTG-AAACAGct</td><td>1</td><td>1</td><td></td></tr>
<tr><td>hsa-miR-99b*</td><td>16,2596</td><td>125</td><td>146</td><td>gccUGGGUGUCUGUGCUCGAAc
   |:::|:|| |:|||:||
ataATTTATAGTAATGAGTTTa</td><td>2</td><td>1</td><td></td></tr>
<tr><td>hsa-miR-567</td><td>16,1778</td><td>68</td><td>90</td><td>caagacaggaccUUCUUGUAUGa
            ||||||||:|
gcaatttagaaaAAGAACATGCa</td><td>1</td><td>1</td><td></td></tr>
<tr><td>hsa-let-7g</td><td>16,1459</td><td>22</td><td>42</td><td>uuGACAUGUUUGAUGAUGGAGU
  || |: |||| :||:|||:|
tcCTCTG-AAACAGCTGCCTTA</td><td>6</td><td>2</td><td></td></tr>
</table>

<table>
<tr><td>hsa-miR-199a-3p</td><td>16,1459</td><td>99</td><td>120</td><td>auuggUUACACGUCUGAUGACA
     ||| |||| | ||||||
ttctgAATTTGCAAAGTACTGT</td><td>5</td><td>1</td><td></td></tr>
<tr><td>hsa-miR-26a-1*</td><td>16,1459</td><td>106</td><td>129</td><td>gcACG-UUCAUUG-GUUCUUAUcc
  ||| ||||| : :|||||||
ttTGCAAAGTACTGTAAGAATAat</td><td>1</td><td>1</td><td></td></tr>
<tr><td>hsa-miR-26a-2*</td><td>16,1459</td><td>107</td><td>129</td><td>cuuugUUCAUUA-GUUCUUAUcc
     ||||| | :|||||||
ttgcaAAGTACTGTAAGAATAat</td><td>1</td><td>2</td><td></td></tr>
<tr><td>hsa-miR-548a-3p</td><td>16,0322</td><td>76</td><td>96*</td><td>CGUUUUCAUUAACGGUCAAAac
| ||||| | || ||||||
GAAAAAGAACATG-CAGTTTga</td><td>5</td><td>1</td><td></td></tr>
<tr><td>hsa-miR-590-5p</td><td>16,0322</td><td>124</td><td>145</td><td>gacgUGAAAAUACUUAUUCGAG
    |:|| || |||:||:|:
aataATTTATAGTAATGAGTTT</td><td>5</td><td>1</td><td></td></tr>
<tr><td>hsa-let-7i</td><td>15,9185</td><td>21</td><td>42</td><td>uugucgugUUUGAUGAUGGAGU
        |||| :||:|||:|
ctcctctgAAACAGCTGCCTTA</td><td>6</td><td>1</td><td></td></tr>
<tr><td>hsa-miR-26a-2*</td><td>15,9185</td><td>232</td><td>253</td><td>cuUUGUUCAUUAGUUCUUAUcc
  ||| ||| |||||||
tcAACTTTGAATAAAGAATAct</td><td>1</td><td>2</td><td></td></tr>
<tr><td>hsa-miR-137</td><td>15,8384</td><td>119</td><td>141</td><td>gAUGCGCAUAAGAAUUCGUUAUU
 || | || |:| ||:|||:|
gTAAGAATAATTTATAGTAATGA</td><td>6</td><td>2</td><td>Down</td></tr>
<tr><td>hsa-miR-217</td><td>15,8384</td><td>71</td><td>93</td><td>agguuagucaaggacUACGUCAu
               |||||||
atttagaaaaagaacATGCAGTt</td><td>5</td><td>2</td><td></td></tr>
<tr><td>hsa-miR-34c-5p</td><td>15,8384</td><td>147</td><td>167</td><td>cgUUAGUCGAUUGAUGUGACGGA
  ||||| || :| :|:|||||
aaAATCAACT—TTTTATTGCCT</td><td>5</td><td>1</td><td></td></tr>
<tr><td>hsa-miR-10a*</td><td>15,8047</td><td>184</td><td>205</td><td>auaAGGGGAUCUAUGCUUAAAc
   |::: || ::||||||
gtgTTTTGTACCAGTGAATTTt</td><td>1</td><td>3</td><td></td></tr>
<tr><td>hsa-miR-200a*</td><td>15,8047</td><td>105</td><td>126</td><td>aGGUCGUGACAGGCCAUUCUac
 :: ||| || | ||||||
aTTTGCAAAGTACTGTAAGAat</td><td>1</td><td>1</td><td></td></tr>
<tr><td>hsa-miR-625*</td><td>15,8047</td><td>116</td><td>137</td><td>acuccccCUUUCAAGAUAUCAg
       ||| |:||||||
actgtaaGAATAATTTATAGTa</td><td>1</td><td>1</td><td></td></tr>
<tr><td>hsa-miR-200b</td><td>15,691</td><td>202</td><td>223</td><td>AGUAGUAAUGGUCCGUCAUAau
|: |:: |: | |||||||
TTTTTGCAATAATGCAGTATgg</td><td>4</td><td>1</td><td></td></tr>
<tr><td>hsa-miR-219-1-3p</td><td>15,691</td><td>221</td><td>239</td><td>GCCCUGCAGGUCUGAGUUGAGA
:|| || |::   :||||||:|
TGGTACATTT---TTCAACTTT</td><td>3</td><td>2</td><td></td></tr>
<tr><td>hsa-miR-382</td><td>15,691</td><td>111</td><td>132</td><td>gcUUAGGUGGUGCUUGUUGAAg
  |:| | :| |||:||:||
aaAGTACTGTAAGAATAATTTa</td><td>1</td><td>2</td><td></td></tr>
<tr><td>hsa-miR-501-5p</td><td>15,5773</td><td>230</td><td>251</td><td>AGAGUGGGUCCCUGUUUCCUAa
|:||| |: | |:|||| ||
TTTCAACTTTGAATAAAGAATa</td><td>2</td><td>1</td><td></td></tr>
<tr><td>hsa-miR-801</td><td>15,5373</td><td>50</td><td>73</td><td>cagcuaaggCGUGCGUCUCGUUAG
         |:|| |:|||||:
gaacctcgaGTACTGTGGGCAATT</td><td>4</td><td>1</td><td></td></tr>
<tr><td>hsa-miR-137</td><td>15,499</td><td>191</td><td>213</td><td>gAUGCGCAUAAGAAUUCGUUAUU
 ||| ||: :|| |||||||
gTACCAGTGAATTTTTGCAATAA</td><td>5</td><td>2</td><td>Down</td></tr>
<tr><td>hsa-miR-10b*</td><td>15,4636</td><td>87</td><td>110</td><td>uaaggGGAUCUUA--GCUUAGACa
     ::|: ||| :||||:||
tgcagTTTGAAATTCTGAATTTGc</td><td>2</td><td>1</td><td></td></tr>
<tr><td>hsa-miR-10a*</td><td>15,3499</td><td>88</td><td>109</td><td>auaaggggaucuAUGCUUAAAC
            | :|||||||
gcagtttgaaatTCTGAATTTG</td><td>2</td><td>3</td><td></td></tr>
<tr><td>hsa-miR-518d-3p</td><td>15,3173</td><td>170</td><td>191</td><td>cGAGGUU-UCCCUUCGCGAAAC
 | |||: | |||:|:||||
tCACCAGCTGCAAAGTGTTTTG</td><td>6</td><td>1</td><td></td></tr>
<tr><td>hsa-miR-10a*</td><td>15,1225</td><td>125</td><td>146</td><td>auaaGGGGAUCUAUGCUUAAAc
    ::: ||| |:||:|||
ataaTTTATAGTAATGAGTTTa</td><td>1</td><td>3</td><td></td></tr>
<tr><td>hsa-miR-126</td><td>15,1225</td><td>206</td><td>228</td><td>GCGUAAUAAUG-AGUGCCAUGcu
:||| || |:| :|:|||||
TGCAATAATGCAGTATGGTACat</td><td>6</td><td>1</td><td></td></tr>
<tr><td>hsa-miR-518a-3p</td><td>14,8951</td><td>169</td><td>191</td><td>aGGUCGUU-UCCCUUCGCGAAAg
 :|| ||: | |||:|:|||
cTCACCAGCTGCAAAGTGTTTTg</td><td>6</td><td>1</td><td></td></tr>
</table>

hsa-miR-518c	14,8202	169	191	ugugagauuucucUUCGCGAAAC<br>|||:|:|||| ctcaccagctgcaAAGTGTTTTG	6	1	
hsa-miR-518e	14,7457	171	190	GUGAGACUUCCCUUCGCGAAA<br>||| | | | |||:|:||| CAC-CAGCTGCAAAGTGTTTT	6	1	
hsa-miR-219-5p	14,6314	54	74	ucuuaacgcAAACCUGUUAGu<br>| |||:||||:<br>ctcgagtacTGTGGGCAATTt	2	1	Down
